# Breed-Specific Dental Variations in Dogs Assessing Malocclusions Using Computed Tomography (CT)

**DOI:** 10.3390/vetsci13050481

**Published:** 2026-05-16

**Authors:** Hamza Habib, Mumta Soothar, Xiaoxuan Pan, Mingfei Ding, Chengli Zheng, Ming Zhang, Ziyao Zhou

**Affiliations:** 1 College of Veterinary Medicine, Sichuan Agricultural University, Chengdu 611130, China; hamzahabib451@gmail.com (H.H.); drmumta954@gmail.com (M.S.); s562751969@163.com (X.P.); 17788369944@163.com (M.D.); 2 Wangmiaotai Animal Hospital, Chengdu 610065, China; 3 Sichuan Institute of Musk Deer Breeding, Chengdu 611731, China; zhengcl@scidc.org.cn; 4 Sichuan Institute for Drug Control, Chengdu 611731, China; 5 College of Animal Science and Technology, Sichuan Agricultural University, Chengdu 611130, China; 6 Key Laboratory of Agricultural Bioinformatics, Ministry of Education, Sichuan Agricultural University, Chengdu 611130, China

**Keywords:** computed tomography (CT), malocclusion in dogs, breed-specific dental deformities, finite element analysis

## Abstract

Dental malocclusions are common and often underdiagnosed situations in dogs, which might result in oral trauma, impaired mastication, and periodontal disease. Nevertheless, scientific investigations into breed-specific variations in dentition remain scarce. To evaluate breed-specific dental variations in dogs, a retrospective cross-sectional analysis was conducted on 92 clinical canine head computed tomography (CT) scans obtained in Chengdu, China, representing a range of breeds and skull morphologies. Dental alignment and occlusal relationships were calculated using standardized malocclusion classification criteria. As a result, malocclusions were found and identified in 46.7% of dogs. Among them, brachycephalic breeds indicated a high prevalence of malocclusion, with Shiba Inu dogs demonstrating the highest malocclusion rate (66.7%), whereas Golden Retrievers and Akitas showed the lowest prevalence (16.7%). Class I malocclusions characterized by dental crowding were most common (44.19%), followed by Class II malocclusions (overbite) (30.23%), and Class III malocclusions (underbite) (20.93%). Our findings demonstrated a strong association between skull morphology and dental alignment abnormalities.

## Simple Summary

Dental problems in dogs, particularly misalignment of teeth, are common but often not noticed during routine examinations. These conditions can lead to pain, difficulty eating, and long-term oral health issues. This study aimed to investigate how dental alignment varies among different dog breeds and skull shapes. We examined head scans from 92 dogs using advanced imaging that provides detailed three-dimensional views of the teeth and jaws. The results indicated that nearly half of the dogs had some form of tooth misalignment. Dogs with shorter and wider skulls were more likely to be affected, while dogs with longer skulls showed less problems. Therefore, small and flat-faced breeds were mostly at higher risk. These findings propose that traditional examination methods miss important dental problems and that more advanced imaging can improve detection. Understanding these differences is important for early diagnosis, better treatment, and improving the overall health and welfare of dogs. This study also highlights the need for more careful breeding practices and routine dental screening, mainly in breeds that are more prone to these conditions.

## 1. Introduction

Dental health is a critical determinant of canine health and wellbeing, as it exerts a profound influence on essential physiological functions including mastication, feeding, and systemic homeostasis [1]. However, dental disorders such as malocclusions and tooth misalignments are frequently underdiagnosed in clinical settings, which may lead to oral trauma and periodontal disease [1]. These dental deformities give rise to severe health complications, including periodontal pathologies, oral soft tissue injuries, and impaired feeding behavior—conditions that are disproportionately prevalent in specific dog breeds selectively bred for extreme anatomical phenotypes [2,3]. Dental malocclusions in dogs are typically classified into four categories, Class I (individual tooth malposition with normal jaw length), Class II (mandibular distoclusion/overbite), Class III (mandibular mesioclusion/underbite), and Class IV (asymmetrical malocclusion). This classification system provides a framework for understanding the clinical consequences of malocclusion across different breeds. However, scientific investigations into breed-specific variations in dentition—particularly those focusing on the prevalence of malocclusions and jaw skeletal morphology—remain scarce [4,5].

Computed tomography (CT) provides superior three-dimensional visualization of dental and craniofacial structures compared with conventional dental radiography [6]. Specifically, CT imaging enables the acquisition of precise, high-resolution three-dimensional (3D) visualizations of dental and maxillofacial structures, eliminating the tissue overlapping, artifacts, and geometric distortion inherent to traditional radiography [7].

While canine dental malocclusions have been extensively documented in the global literature [8], studies focusing on dog populations in China, especially those in Chengdu, remain relatively limited. This research gap has resulted in a dearth of region-specific data regarding breed-related dental variations. The present study aims to investigate the relationship between selective breeding and dental morphological variations, with a particular focus on the underlying mechanisms involving genetic predispositions, developmental constraints, and disproportionate cranial skeletal development.

## 2. Materials and Methods

### 2.1. Study Design

This retrospective cross-sectional study was conducted over a period from December 2024 to May 2025, which included 92 clinical canine head CT scans representing a diverse range of breeds and skull morphologies. Dogs were categorized by skull type as mesocephalic (*n* = 66), brachycephalic (*n* = 25), and dolichocephalic (*n* = 1) based on established cranial classification criteria [7]. CT scans were selected based on availability of complete craniofacial imaging data, confirmed absence of head-related diseases as examined by professional veterinary radiologists.

### 2.2. CT Scan Data Acquisition

CT scans of the dogs’ skulls were acquired using various high-resolution imaging systems. Scans were collected from multiple veterinary facilities across Chengdu, including the College of Veterinary Medicine at Sichuan Agricultural University, China. The scans were performed for both specific clinical concerns and routine screening. In some cases, the scans were conducted to assess cranial morphology and malocclusions in dogs with suspected anatomical variations, while in other cases, routine screening was performed as part of the general veterinary assessment. No exclusion criteria were applied, as the animals selected for the study did not have any specific health conditions that would impact the analysis of cranial morphology and malocclusions. Imaging parameters were standardized to ensure diagnostic quality and consistency across datasets. Head positioning during imaging was standardized using a head holder to ensure proper alignment and minimize variability. Scans with a voxel resolution of 0.3 mm × 0.3 mm × 0.3 mm and equivalent high-resolution settings were prioritized for precise measurement of tooth dimensions and malocclusion assessment. All CT scan data were provided in DICOM format for three-dimensional reconstruction and morphometric analysis [9].

### 2.3. Image Processing and 3D Reconstruction

**Figure 1:**
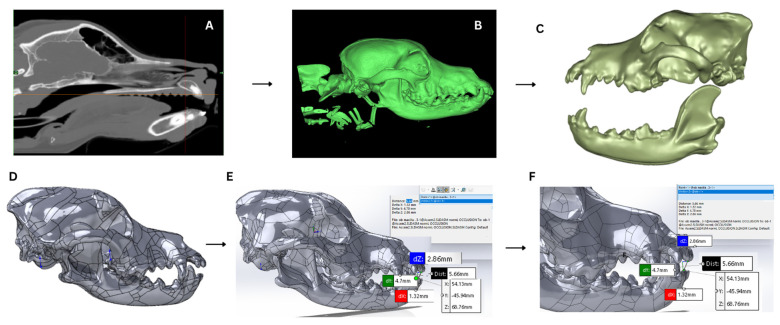
CT-based workflow for evaluation of occlusal relationships in dogs. (**A**) Sagittal CT image. (**B**) 3D skull reconstruction. (**C**) Separated maxilla and mandible. (**D**) Mesh model. (**E**) Occlusal measurement. (**F**) Detailed view of horizontal occlusal measurement.

CT-based morphometric assessment was performed using three-dimensional reconstructed images to evaluate dental alignment and occlusal relationships. All CT scans were imported in DICOM format in Mimics Medical (Materialise Co., Ltd., Leuven, Belgium, Version 21.0.406), and three-dimensional reconstruction was completed earlier to measurement. Segmentation was performed using predefined threshold range 1500 HU (minimum) to 2896 HU (maximum) using the ″New Mask″ tool to isolate mineralized dental tissues [9]. The threshold histogram was visually inspected to check proper separation of mineralized tissues, and the ″Fill holes″ tool was started to improve mask continuity. After initial segmentation, the ″Edit Mask″ tool was used to remove artifacts and refine the region of interest, with the lasso tool applied in erase mode to eliminate unwanted structures. Three-dimensional polygonal models of the skull and dentition were separated using the ″Region Grow function″ by selecting connected voxel regions within the defined mask. After mask refinement, the ″Calculate Part″ tool was used to generate three-dimensional polygonal models. The reconstruction quality was set to High, with interpolation based on contour and continuity preference selected to preserve anatomical smoothness. Surface smoothing was limited to minimal iterations (2 iterations and smooth factor 0.5) to maintain geometric fidelity. The reconstructed models were visually inspected for anatomical accuracy and mesh continuity (Figure 1).

### 2.4. Occlusal Measurement and Morphometric Analysis

Occlusal relationships and malocclusion patterns were calculated using multiplanar CT reconstructions, with particular emphasis on sagittal views to assess the craniocaudal relationship between the maxilla and mandible. The horizontal occlusal relationship (overjet) was measured as the linear distance between the incisal tips of the maxillary and mandibular incisors using the ″Measure Distance″ tool in the SOLIDWORK 2026 software. Measurements were obtained on appropriately aligned sagittal slices to approximate the natural occlusal relationship. Multiplanar reconstruction (axial, sagittal, and coronal views) was used to confirm accurate landmark identification and ensure consistency of measurements. Because CT scans were obtained under anesthesia with endotracheal intubation, occlusion was assessed based on the relative anatomical alignment of maxillary and mandibular teeth and skeletal jaw relationships rather than direct tooth contact. The spatial relationship between maxillary and mandibular incisors and canine teeth was examined to determine occlusal alignment. Each measurement was repeated twice, and the mean value was recorded to reduce measurement error.

### 2.5. Malocclusion Classification and Prevalence Analysis

Based on the above measurements, malocclusions were classified based on the American Veterinary Dental College (AVDC) standards [10]. The prevalence of malocclusion types was compared among different skull types (brachycephalic, mesocephalic, and dolichocephalic) [11]. Dogs were grouped by breed, skull type, and body size for comparative prevalence analysis. Breed-specific malocclusion prevalence was calculated using the formula [12]:

Prevalence (%) = (Number of dogs with malocclusion in a breed/Total scans for that breed) × 100

### 2.6. Statistical Analysis

All statistical analysis in the following sections were conducted using Excel and SPSS v27 (IBM, New York, NY, USA), with a significance threshold of *p* < 0.05 was considered as statistically significant [9]. Descriptive statistics were used to summarize the data. The Chi- square test of independence was performed to calculate the relationship between variables including breed and skull morphology with the presence of malocclusion.

## 3. Results

### 3.1. Description of CT Data and Malocclusion

A total of 92 canine head CT scans were analyzed, all of which were suitable for detailed assessment of craniofacial morphology and dental occlusion. The dataset comprised 43.43 GB of high-resolution DICOM data, with each scan consisting of multiple contiguous axial slices suitable for three-dimensional craniofacial analysis. Malocclusion was identified in 43 cases, resulting in an overall prevalence of 46.7% among the examined dogs. The study population included 50 males (54.3%) and 42 females (45.7%). The mean age of the dogs was 6.73 ± 3.69 years (range: 1–14 years). The majority of dogs showed mixed dentition, indicating the presence of both deciduous and permanent teeth during the period of dental development.

### 3.2. Breed-Wise Variations in Malocclusion Prevalence

Breed-based analysis revealed modifications in malocclusion frequency between breeds signifying the role of genetic and craniofacial elements. Shiba Inus and Yorkshire Terriers were the most affected (66.7%), followed by French Bulldogs, Bichons, and Pomeranians (60–62.5%).

**Figure 2:**
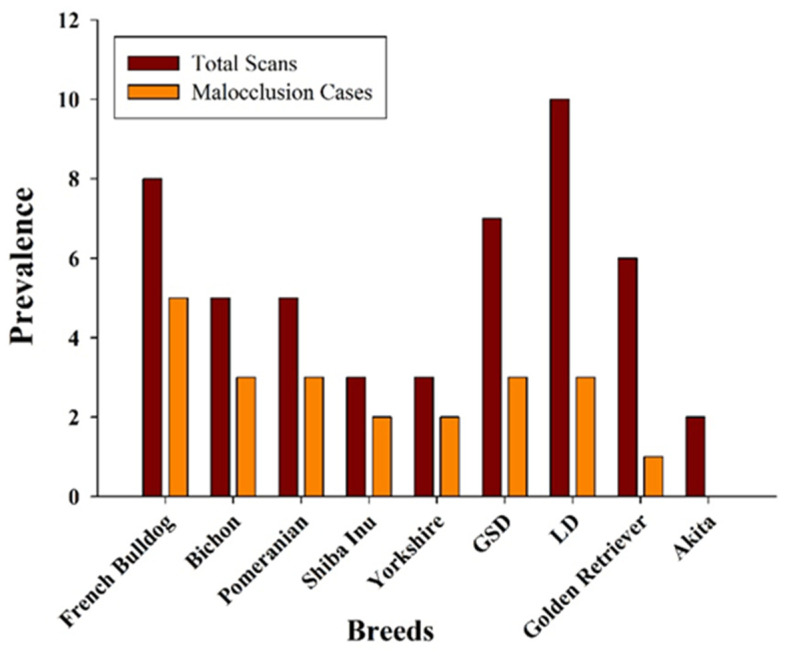
Comparative analysis of malocclusion prevalence across various dog breeds. GSD: German Shepherd Dog; LD: Labrador Dog.

In contrast, Golden Retrievers (16.7%) showed the lowest prevalence, while no malocclusion cases were observed in Akita (*n* = 1). A Chi-square test was performed to calculate the relationship between breed and malocclusion prevalence (x^2^ = 5.87, df = 8, *p* = 0.66) were not statistically significant due to small sample sizes. Labrador, Golden Retriever, and German Shepherd Dog indicated mixed malocclusions which represented the variety of malocclusion prevalence even among similar breed categories (Figure 2).

### 3.3. Influence of Skull Morphology on Malocclusion Prevalence

Skull type analysis revealed differences in the prevalence of malocclusions across canine cranial classification categories. Brachycephalic breeds exhibited the highest susceptibility, with 65% (13/20) affected; this rendered them 1.6 times more likely to develop malocclusions compared to mesocephalic breeds (41.4%, 29/70). Although mesocephalic breeds were less predisposed to malocclusions than brachycephalic breeds, they still demonstrated a substantial prevalence (41.4%), indicating that occlusal abnormalities are not exclusive to extreme skull morphologies. No malocclusion cases were detected in dolichocephalic breeds (0/1). A Chi-square test was performed to calculate the relationship between skull morphology and malocclusion prevalence with results (x^2^ = 5.20, df = 2, *p* = 0.074) not statistically significant due to small sample sizes. However, the small sample size of this group limits the definitiveness of this finding. These results underscore a strong correlation between skull morphology and malocclusion risk, emphasizing the need for breed-specific dental monitoring—particularly in brachycephalic populations, where preventive care can mitigate severe clinical outcomes.

### 3.4. Distribution and Prevalence of Malocclusion Classes

**Figure 3:**
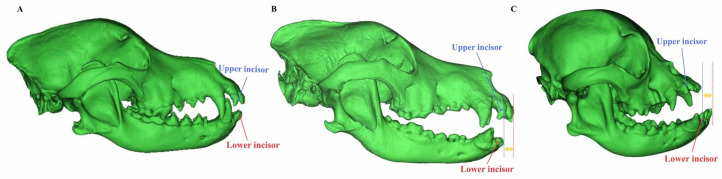
Representative sagittal CT reconstructions illustrating occlusal relationships in canine skulls. (**A**) Normal occlusion with appropriate maxillomandibular alignment. (**B**) Class II malocclusion (overbite) characterized by posterior displacement of the mandible relative to the maxilla. The lower incisor is positioned caudal to the upper incisor (yellow arrow indicates the horizontal overjet). (**C**) Class III malocclusion (underbite) characterized by anterior displacement of the mandible relative to the maxilla. The lower incisor is positioned rostral to the upper incisor (yellow arrow indicates the horizontal underjet).

Analysis of malocclusion classes revealed distinct patterns across canine breeds. Class I malocclusions were the most prevalent, accounting for 44.19% of all malocclusion cases and 20.65% of total scans. This malocclusion type predominantly affected small breeds, including Bichons and Pomeranians, and was characterized by dental crowding and tilted teeth. The mandibular incisor was the most prevalent affected tooth in Class 1 malocclusion, occurring in cases and demonstrating the prevalence of angular and tilted teeth in these cases. Class II malocclusions, representing 30.23% of cases and 14.13% of total scans, were commonly observed in mesocephalic breeds such as Labradors, Retrievers, and German Shepherds, typically presenting with overbites. Class III malocclusions, which constituted 20.93% of cases and 9.78% of total scans, exhibited strong breed specificity, and included dental crowding and displacement of mandibular canine teeth exclusively in brachycephalic dogs (e.g., French Bulldogs) and manifesting as severe Class III malocclusions. The sagittal CT reconstructions illustrating occlusal relationships in canine skulls in (Figure 3). The rare Class IV malocclusions, accounting for approximately 4.65% of cases, were restricted to isolated instances of dental spacing abnormalities. The distribution and prevalence of malocclusion classes are summarized in (Table 1).

## 4. Discussion

This study highlighted a malocclusion prevalence of 46.7% based on CT imaging emphasizing an important concern for canine dental health. Traditional diagnostic methods including routine oral examination and conventional radiography, which has previously been reported to range from (10–30%) [12], may underestimate the true incidence of dental misalignments in dogs. Our findings suggest that malocclusion may be more common in the general canine population than previously recognized, but further studies are needed to confirm this trend.

### 4.1. Prevalence and Patterns of Malocclusion

Class I malocclusions described by dental crowding without skeletal anomalies were the most prevalent, signifying 44.19% of all malocclusion cases. These findings align with the results that dental crowding and premature tooth loss are common in toy breeds due to restricted mandibular growth relative to dental arch requirements [5]. Class II malocclusion observed in 14.13% of total dogs was mainly present in mesocephalic breeds including Labradors and German Shepherds. Although these breeds generally have more proportionate cranial structures, overbites may be influenced by developmental differences between maxillary and mandibular growth. This type of malocclusion has been related with genetic drift in non-brachycephalic breeds in his broader exploration of jaw-dental relationships [13]. These results highlight findings that Class III malocclusions were a hallmark of brachycephalic morphology, where maxillary hypoplasia results in forward protrusion of the lower jaw [3].

Our findings corroborate previous data indicating malocclusion prevalence ranging from (30–70%) [14], suggesting that selective breeding practices aimed at enhancing appearance often inadvertently increase the risk of dental misalignments and welfare implications of prioritizing craniofacial aesthetics over functional anatomy. These genetic selection may result in dogs with skulls that lack the proportional space to accommodate normal dentition thus driving up malocclusion rates [3,4].

### 4.2. Influence of Skull Morphology on Malocclusion Risk

The previous literature has acknowledged an association between skull morphology and malocclusion risk but lacks computed tomography (CT)-based quantitative data [15]. Our findings bridge the gap between prior hypotheses and novel evidence via CT imaging that brachycephalic breeds exhibit a 65% malocclusion rate higher than that of mesocephalic (41.4%) and dolichocephalic (0%) breeds. This result corroborates previous studies demonstrating that skull compression in brachycephalic breeds constrains dental arch space [7,14].

Our finding supports prior research indicating that elongated jaw structures facilitate natural tooth eruption by offering sufficient spatial accommodation, thereby reducing the likelihood of malalignment [4,12]. The proportionate jaw development in dolichocephalic skulls promotes proper occlusion through adequate spatial provision for dentition [16]. Additionally, our results reveal that skull type is a more robust predictor of malocclusion risk than breed or body size. Specifically, mesocephalic breeds with similar body sizes exhibited distinct malocclusion patterns, suggesting that cranial shape alone exerts a significant regulatory effect on occlusal outcomes [5]. These results are consistent with previous findings that cranial morphology plays a more definitive role in dental alignment than breed classification per se, and further validate the heightened susceptibility of brachycephalic breeds to Class I and III malocclusions [3]. This confirms that extreme craniofacial selective breeding often results in severe anatomical compromises. Moreover, the brachycephalic skull architecture not only disrupts dental alignment but also impairs orbital structure and cranial function, with substantial implications for canine health and welfare [14]. Although no malocclusions were observed in dolichocephalic dogs in the present study, the small sample size precludes definitive conclusions; even so, this finding supports previous recommendations that elongated jaw structures provide greater dental arch space, thereby minimizing the risk of occlusal conflict [15]. Given the strong association between skull type and malocclusion, early preventive dental care should prioritize brachycephalic and small breeds. Incorporating skull-type evaluation into routine screenings, coupled with genetic counseling for breeders, may help mitigate structural dental abnormalities. A limitation of this study is the lack of direct evaluation of dental occlusion. The study focuses mainly on anatomical and structural factors, such as cranial morphology, rather than the functional relationship between the teeth. While this approach provides valuable insights into the underlying anatomical causes of malocclusion, it does not fully capture the functional aspects of dental misalignment. However, the findings remain significant in highlighting the role of cranial structure in malocclusion risk. Future studies that combine structural imaging with direct occlusion evaluation could provide a more comprehensive understanding.

### 4.3. Factors Which Might Contribute to Malocclusion

The high prevalence of malocclusion detected in this study (46.7%) is mainly among brachycephalic and small breeds, which might be attributed to a combination of genetic, environmental, dietary, and breeding-related factors. According to our results in which French Bulldogs, Pomeranians, and Shiba Inus showed malocclusion rates of over 60%. Malocclusion is often genetic with disproportionate jaw size and mismatches between tooth and jaw dimensions that are common in breeds like Bulldogs, Dachshunds, and Chihuahuas [12,17]. According to the references, genetic mutation in SMOC2, BMP3, and FGF4 have been associated in Class III malocclusions and Class II malocclusions [18]. While genes like PAX9 and MSX1 regulate tooth development and are associated with specific malocclusion Class II [19,20], the genetic mechanisms underlying malocclusion were not directly explored in this study. Future research could delve deeper into the genetic basis of these malocclusions to provide a more comprehensive understanding of their role in breed-specific dental variations. Additionally, dogs that are not exposed to appropriate chewing stimuli due to soft food diets, oral dysfunction, and breathing issues could experience reduced muscular stimulation and underdeveloped jaw strength, which have been linked to increase malocclusion rate [21]. Nutritional imbalances including calcium-phosphorus ratios and vitamin D deficiency have also been linked to uneven jaw development [22,23]. This study did not examine the influence of diet and chewing habits. Future research could explore this hypothesis to better understand its potential impact on malocclusion development.

Our findings regarding the high malocclusion rates in purebred populations (e.g., French Bulldogs and Pomeranians) are supported by genomic studies demonstrating that these breeds are more susceptible to inherited dental deformities [23,24]. Breeding practices aimed at preserving breed-specific phenotypic traits often compromise genetic health, with increased homozygosity contributing to both dental crowding and jaw abnormalities [24,25]. The interplay of genetic, environmental, and lifestyle-related factors underscores the urgent need for preventive measures, reforms in selective breeding practices, and early diagnostic interventions—particularly in high-risk canine populations. The presence of additional teeth was not calculated in this study, as the main focus was on occlusal relationships and tooth alignment. Future studies incorporating tooth number variations may provide further insights into canine dental abnormalities.

## 5. Conclusions

This study confirms that skull morphology plays a pivotal role in shaping dental traits and determining malocclusion risk across dog breeds. Consequently, the elevated malocclusion rates observed in brachycephalic and small breeds highlight the anatomical consequences of selective breeding for extreme craniofacial traits. These results advocate for the implementation of breed- and skull-type-specific dental screening protocols in clinical veterinary practice. Incorporating advanced imaging techniques, alongside targeted preventive care and early detection strategies, enhance the management of dental issues in high-risk canine populations. CT imaging provided detailed visualization of dental alignment and structural abnormalities, allowing identification of malocclusion patterns that were not consistently evident on routine examination. Breed-specific variation in malocclusion prevalence was observed during this study. Such efforts will ultimately contribute to improved oral health and overall welfare in high-risk canine populations.

## Figures and Tables

**Table 1:** GSD = German Shepherd Dog; Lab = Labrador Retriever. Overall prevalence is based on total scans (*n* = 92), and prevalence among malocclusions is based on total cases (*n* = 43).

Class	Cases	Prevalence (Overall)	Prevalence (Among Malocclusions)	Breeds
Class 1	19	20.65% (19/92)	44.19% (19/43)	Bichon, GSD, Shiba Inu
Class 2	13	14.13% (13/92)	30.23% (13/43)	Lab, Pomeranian, Yorkshire, Golden Retrievers
Class 3	9	9.78% (9/92)	20.93% (9/43)	French Bulldog, Bichon
Class 4	2	2.17% (2/92)	4.65% (2/43)	French bulldog

## Data Availability

The original contributions presented in the study are included in the article, further inquiries can be directed to the corresponding authors.

## Abbreviations

The following abbreviations are used in this manuscript:

CT	Computed Tomography
STL	Stereolithography file format
AVDC	American Veterinary Dental College
